# Disentangling malaria anaemia

**DOI:** 10.1186/1475-2875-13-S1-O21

**Published:** 2014-09-22

**Authors:** Kathrin Schuldt, Jennifer Evans, Julia Lenzen, Christa Ehmen, Birgit Muntau, Jurgen Sievertsen, Yeetey Enuameh, Kingsley Osei-Kwakye, Ernest Cudjoe Opoku, Edmund NL Browne, Rolf Horstmann, Christian Timmann

**Affiliations:** 1 Bernhard Nocht Institute for Tropical Medicine, Hamburg, Germany; 2 Kumasi Centre for Collaborative Research in Tropical Medicine, Kumasi, Ghana; 3 University of Copenhagen, Copenhagen, Denmark; 4 Kwame Nkrumah University of Science and Technology, Kumasi, Ghana

## Background

Anaemia is a major health burden in sub-Saharan Africa. With an estimated two-thirds of children being affected, childhood anaemia causes poor cognitive development, reduced growth and impaired immune functions associated with increased mortality. In malaria-endemic regions, particularly in those with perennial *Plasmodium falciparum* transmission, most of the childhood anaemia disease burden is being attributed to malaria. The pathogenesis of malaria anaemia, which may well be the single most frequent cause for childhood mortality worldwide, has been debated for many decades.

## Materials and methods

In order to identify underlying mechanisms for malaria anaemia two studies were evaluated: a cohort study including 465 children that were followed weekly over eight months and a severe malaria case-control study including >4,500 children, both carried out in a hyperendemic malaria area of Ghana, West Africa. Human genetic polymorphisms of candidate genes were genotyped and tested for association with malaria anaemia in both studies.

## Results

We found several genotype-phenotype associations, which had differing effects after stratifying for anaemia with and without complications. For instance, uncomplicated anaemia was found associated with the A allele of rs196854, which was associated previously with an increased expression of interleukin-6, a key mediator of the systemic inflammatory response.

## Conclusions

Our results suggest that there are at least two different underlying mechanisms of malaria anaemia, uncomplicated anaemia characterized by a slow and continuous development towards a deficit of red blood cells, and anaemia with complications characterized by a rapid and substantial loss of red blood cells. The uncomplicated form appears to be caused by chronic systemic inflammatory processes rather than malaria episodes or parasite densities, similar to what is found in other forms of anaemia of chronic disease (ACD).

